# Supplementary material for: Novel Driver Strength Index highlights important cancer genes in TCGA PanCanAtlas patients
Source: PeerJ. 2022 Aug 11;10:e13860. doi: 10.7717/peerj.13860 (PMC9375969; doi:10.7717/peerj.13860)
Supplement: Supplemental Information 2 [file peerj-10-13860-s002.zip › COHORTS/genes plots/cohorts/2021_8_16_14_9_distribution_cohorts_LIHC.pdf]

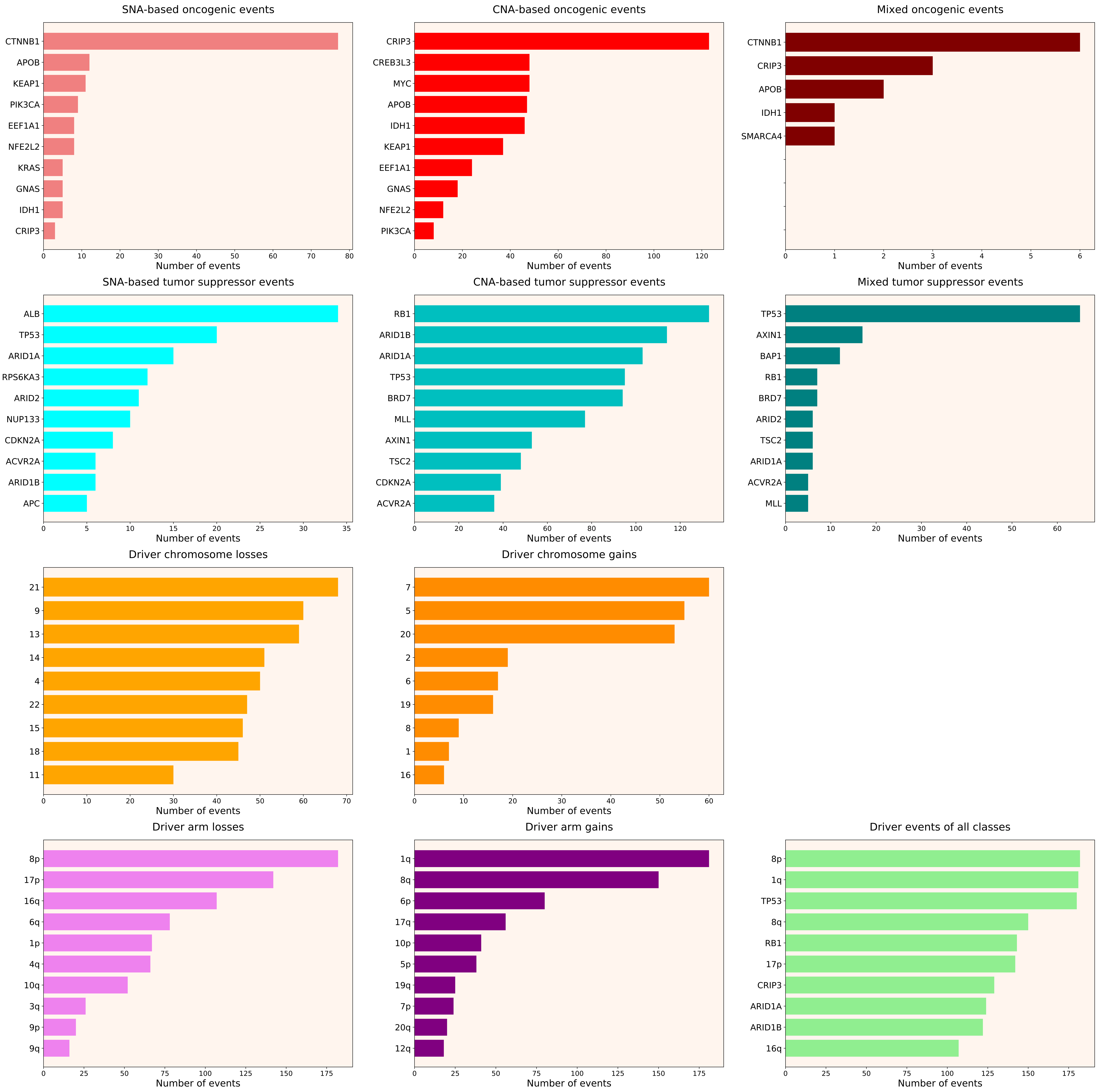
SNA-based oncogenic events
CTNNB1
APOB
KEAP1
PIK3CA
EEF1A1
NFE2L2
KRAS
GNAS
IDH1
CRIP3
0 10 20 30 40 50 60 70 80
Number of events
CNA-based oncogenic events
CRIP3
CREB3L3
MYC
APOB
IDH1
KEAP1
EEF1A1
GNAS
NFE2L2
PIK3CA
0 20 40 60 80 100 120
Number of events
Mixed oncogenic events
CTNNB1
CRIP3
APOB
IDH1
SMARCA4
0 1 2 3 4 5 6
Number of events
SNA-based tumor suppressor events
ALB
TP53
ARID1A
RPS6KA3
ARID2
NUP133
CDKN2A
ACVR2A
ARID1B
APC
0 5 10 15 20 25 30 35
Number of events
CNA-based tumor suppressor events
RB1
ARID1B
ARID1A
TP53
BRD7
MLL
AXIN1
TSC2
CDKN2A
ACVR2A
0 20 40 60 80 100 120
Number of events
Mixed tumor suppressor events
TP53
AXIN1
BAP1
RB1
BRD7
ARID2
TSC2
ARID1A
ACVR2A
MLL
0 10 20 30 40 50 60
Number of events
Driver chromosome losses
21
9
13
14
4
22
15
18
11
0 10 20 30 40 50 60 70
Number of events
Driver chromosome gains
7
5
20
2
6
19
8
1
16
0 10 20 30 40 50 60
Number of events
Driver arm losses
8p
17p
16q
6q
1p
4q
10q
3q
9p
9q
0 25 50 75 100 125 150 175
Number of events
Driver arm gains
1q
8q
6p
17q
10p
5p
19q
7p
20q
12q
0 25 50 75 100 125 150 175
Number of events
Driver events of all classes
8p
1q
TP53
8q
RB1
17p
CRIP3
ARID1A
ARID1B
16q
0 25 50 75 100 125 150 175
Number of events
